# Niche Divergence versus Neutral Processes: Combined Environmental and Genetic Analyses Identify Contrasting Patterns of Differentiation in Recently Diverged Pine Species

**DOI:** 10.1371/journal.pone.0078228

**Published:** 2013-10-29

**Authors:** Alejandra Moreno-Letelier, Alejandra Ortíz-Medrano, Daniel Piñero

**Affiliations:** Departamento de Ecología Evolutiva, Instituto de Ecologia, Universidad Nacional Autonoma de México, México D.F., Mexico; Fordham University, United States of America

## Abstract

**Background and Aims:**

Solving relationships of recently diverged taxa, poses a challenge due to shared polymorphism and weak reproductive barriers. Multiple lines of evidence are needed to identify independently evolving lineages. This is especially true of long-lived species with large effective population sizes, and slow rates of lineage sorting. North American pines are an interesting group to test this multiple approach. Our aim is to combine cytoplasmic genetic markers with environmental information to clarify species boundaries and relationships of the species complex of *Pinus flexilis*, *Pinus ayacahuite,* and *Pinus strobiformis*.

**Methods:**

Mitochondrial and chloroplast sequences were combined with previously obtained microsatellite data and contrasted with environmental information to reconstruct phylogenetic relationships of the species complex. Ecological niche models were compared to test if ecological divergence is significant among species.

**Key Results and Conclusion:**

Separately, both genetic and ecological evidence support a clear differentiation of all three species but with different topology, but also reveal an ancestral contact zone between *P. strobiformis* and *P. ayacahuite*. The marked ecological differentiation of *P. flexilis* suggests that ecological speciation has occurred in this lineage, but this is not reflected in neutral markers. The inclusion of environmental traits in phylogenetic reconstruction improved the resolution of internal branches. We suggest that combining environmental and genetic information would be useful for species delimitation and phylogenetic studies in other recently diverged species complexes.

## Introduction

Solving species relationships among recently diverged taxa is a topic that has received a lot of attention in recent times. Shared ancestral polymorphism and gene flow can obscure relationships, greatly limiting our understanding of the diversification processes in many lineages [1]–[4]. This is particularly true for recently diverged species, where most neutral variation is still shared among lineages and reproductive barriers are weak [5], [6]. Especially if speciation is driven by ecological divergence, then neutral markers alone might not be able to reconstruct boundaries and relationships [7]. To overcome this problem, a multiple evidence approach is useful in delimiting species and resolving species relationships [8]–[12]. This approach would help to identify species boundaries and shed light on the speciation process by incorporating genetic information with different coalescent histories and ecological niche information [11]. This would help overcome the problem of non-monophyly of alleles within a species, which is the common pattern seen in many plant groups.

There are several factors that can produce, combined or in isolation, a pattern of non-monophyly of alleles within species: 1) Recent or incomplete speciation; 2) Large effective population sizes; 3) Gene flow and introgression [1], [6]. All of these factors are fairly common in some groups of recently diverged species, such as the outcrossing, long-lived pine trees. North America is a center of diversity of the genus *Pinus*, with several subsections found exclusively in this geographic region [13]–[15]. Most of these species belong to groups of recent divergence like subsections *Cembroides*, *Australes* or *Ponderosae,* whose relationships have been difficult to resolve in molecular phylogenies [16]–[19]. Adding to the recent divergence, in general a significant number of species of the same subsection show no reproductive incompatibility [15] and several cases of hybridization and introgression have been documented [19]–[21]. Thus, it has been difficult to establish the relationships among species and to delimit species using traditional phylogenetic and morphological criteria [16]–[19]. In these problematic cases, a combined approach that includes genealogical data and ecological niche information could help clarify relationships for closely related species [11], [22], [23].

*Pinus ayacahuite* (Ehrenberg ex Schlechtendahl), *Pinus flexilis* (James) and *Pinus strobiformis* (Engelmann), (Subgenus *Strobus*, Section *Quinquefoliae;* [16]) form a species complex (Ayacahuites *sensu* [24]) of probable recent divergence [16]–[18], [25], and share a montane affinity. Each species occupies a distinct and non-sympatric geographic range: *P. flexilis* in the Rocky Mountains of Western United States and Canada; *P. strobiformis* in the Sierra Madre Occidental (SMOCC) and Sierra Madre Oriental (SMOR) in Northern Mexico, as well as some isolated populations in the United States and Central Mexico; *P. ayacahuite* in the Trans-Mexican Volcanic Belt (TMVB), Sierra Madre del Sur and the Highlands of Chiapas and Central America [14]. The wide distribution, high morphological variation, and indirect evidence of hybridisation has led many authors to question the taxonomic status of one or several of the members of this complex [13]–[15], [26]. Previous studies using nuclear genes and chloroplast microsatellites [27], [28] have identified shared polymorphism in these three species. In addition, many of the markers used for plant phylogenetic studies lack enough informative variation (*matK*, *rbcL*, *rps16*, etc.; [16], [21], [25]). This lack of differentiation using molecular data and the possibility of past hybridisation obscure the relationships within the species in this complex using genetic information alone, in particular when only one individual per species is used.

In the present work, we intend to clarify the relationships and boundaries inside this species complex using both genetic markers and ecological niche modeling, and test the role of niche divergence in the speciation process. To do so we implement a genealogical perspective, including several samples from each taxon from their entire geographical range, to account for intraspecific polymorphism. We used a principal component analysis to characterise the ecological niche of the three species, followed by ecological niche modeling (ENM) to test the degree of ecological differentiation of each species. Theory predicts that under ecological speciation, natural selection will promote adaptation to different environments [29], thus niche divergence should be the observed pattern; alternatively, under a allopatric speciation followed by genetic drift, niche conservatism is the expected pattern [30]–[32]. Subsequently, we performed phylogenetic analyses using different combinations of DNA information obtained from chloroplast and mitochondrial genomes as well as environmental information to resolve the relationships within the species complex. This combined approach could allow us to understand the species relationships and the speciation process.

## Materials and Methods

### Ethics Statement

This work did not involve endangered or protected species. Plant tissue sampling was non destructive and did not require special permits from the Secretaria de Recursos Naturales from Mexico, or the United States Forest Service.

### Niche Characterisation, Divergence and Ecological Niche Modeling

Geographical information of species distribution was obtained from both herbaria information and field sampling. For *P. flexilis*, geographical coordinates of species occurrence were obtained from the Missouri Botanical Garden (http://www.tropicos.org/; last visited: 27/Sept/2011) and the Consortium of Pacific Northwest Herbaria (http://www.pnwherbaria.org/); for *P. strobiformis* and *P. ayacahuite,* geographical coordinates were obtained from The World Information Network on Biodiversity (http://www.conabio.gob.mx/remib/doctos/remib_esp.html), Herbario BIGU from the Universidad de San Carlos de Guatemala (M. Veliz; pers. com.), and our own field information. All coordinates were verified and only those with degrees, minutes, and seconds were used. The taxonomic status of each locality was determined following Farjon and Styles [14]. A list of all coordinates used in modeling can be found in the Table S1 in File S1.

Overall niche differentiation was evaluated by a Principal Component Analysis (PCA), using presence points of each species to extract the point values of climatic variables across the 19 bioclimatic layers obtained from the WORLDCLIM database [33]. To improve the detection of correlations between variables and avoid bias due to geographical proximity of presence points [10], [32], we also included in the PCA 1000 random points generated along the entire distribution range of the three species. The PCA axis scores were then used to perform a multivariate analysis of variance (MANOVA) to test the significance of overall niche separation, using species as independent variables. Additionally, pairwise contrast analyses were performed for each species pair (*ayacahuite-flexilis, ayacahuite-strobiformis, strobiformis-flexilis*) to test for differences of environmental variables in between species, and a canonical analysis was used to determine which principal component contributed more to the differences among the species. The analyses were performed with the statistical package JMP 9.0 (SAS Institute Inc. Cary, NC, 1989–2010).

The ecological niche models were done with the software Maxent 3.3.1 [34] with the same bioclimatic variables used in the PCA, for both present and LGM conditions. All settings were set at default. The climatic variable layers for present conditions were obtained from the WORLDCLIM Database and had a resolution of 30 arc seconds (http://www.worldclim.org; last visited: 27/Sept/2011). The Last Glacial Maximum (LGM) climate data was developed by the Community Climate System Model (CCSM) with a resolution of 2.5 arc-minutes, also available at the WORLDCLIM site. The occurrence for each model was graphically represented using a threshold of the lowest probability of occurrence of a presence point: *P. flexilis* (0.21), P. *ayacahuite* (0.45) and *P. strobiformis* (0.14).

To evaluate the ecological niche divergence between pairs of species, we used the background test implemented by ENM Tools 1.0 [35]. We tested niche divergence without assuming any particular relationship between species. This test compares two ecological niche models obtained from two species with partially or non-overlapping distributions to see if they are more similar or different from each other than expected by chance. This test will allow us to identify possible niche conservatism or ecological differentiation that could explain the causes of speciation in the species complex [30], taking into account the environment available for each species, and thus avoiding possible biases towards niche divergence [36]. All analyses were carried only with the inferred ENMs for present conditions.

Two comparisons are run for each case, to evaluate if species A can predict the niche of species B, and vice versa (niche conservatism) estimating a value of niche similarity (*I*) that can go from 0 (no overlap) to 1 (the two niches are identical). If the *I* value of niche similarity is not significantly larger than expected by chance, we would conclude that there is no niche conservatism. The test was run with 100 replicates and was analysed with a two-tailed test [36].

### Genetic Differentiation and Phylogenetic Analyses

Individuals from *P. flexilis*, *P. strobiformis* and *P. ayacahuite* were collected across the entire range of each species following the taxonomic delimitation of Farjon and Styles for practicality ([14]; details of the sampling sites can be found in [28], [37]; and Table S2 in File S1). DNA extraction was carried out using a CTAB protocol [38]. The individuals used in all analyses were chosen at random from distinct geographical areas to ensure an unbiased sampling. Three non-coding regions from the chloroplast were evaluated for intraspecific polymorphism using 5 individuals of each species for a preliminary screening: the spacers of *atpB-rbcL* [39], *trnL-trnF* [40], *trnG-trnS* and the intron of *trnG* [41]. The *atpB-rbcL* spacer was amplified using the conditions reported by Parducci and Szmidt [42], and the remaining regions were amplified using the original reported conditions. The amplicons for all regions were purified using the gel extraction kit QIAquick (Qiagen, Hilden, Germany), and sequenced by the Macrogen sequencing service (http://www.macrogen.com, Seoul, Korea). Once polymorphism was detected, those markers were amplified in the rest of the sample: 16 individuals of *P. flexilis*, 20 of *P. strobifomis* and 26 of *P. ayacahuite*. *Pinus strobus* (accession: FJ899560) and *Pinus lambertiana* (accession: FJ899577) sequences were used as outgroups in all reconstructions and were obtained from complete chloroplast sequences from GenBank (http://www.ncbi.nlm.nih.gov).

For the mitochondrial genome, introns 1 and 4 of *nad5* (*nad5a* and *nad5d*) gene were scanned for polymorphism [43]. The PCR amplification conditions followed those described in Shaw et al. [41]. Only 9 individuals of *P. flexilis* were included because intron 4 of *nad5* could not be amplified in all the samples. The sample size for the other two species was 26 for *P. strobiformis* and 20 for *P. ayacahuite*. Outgroups for mitochondrial markers are the same as for the chloroplast markers and were obtained from GenBank (*P. lambertiana* accession: JN050891 and *P. strobus* accession: JN050948). The sequences from the polymorphic amplicons were concatenated into two different datasets, one for chloroplast sequences and another for mitochondria, and evaluated for genetic diversity. The following metrics were estimated: number of haplotypes (h), number of segregating sites (S), haplotype diversity (H_d_), nucleotide diversity (**π**) and Watterson estimate for population mutation rate (θ_W_). Genetic differentiation between species was estimated using several metrics: H_ST_ and F_ST_ that account for population differentiation, considering the sequences from one species as a populations, and S_nn_ or nearest neighbour statistic, that takes into account the number of substitutions in the sequences. All genetic diversity and genetic differentiation estimates were conducted with DnaSP 5 [44].

Phylogenetic reconstructions were preformed using a tree sampling Bayesian method implemented by BEAST 1.7.3, applying a coalescent tree evolution model [45]. Different types of data were used to reconstruct trees: a) Chloroplast and mitochondrial sequences obtained as described above; b) Chloroplast microsatellites (cpSSR) previously published [28], [37]; c) Environmental information in the form of the first two principal components of the analysis described in the previous section and coded as continuous characters. Chloroplast and mitochondrial sequences were treated as independent partitions. Chloroplast microsatellites were converted to haplotypes and coded as binary characters as described in [46], to ensure they were treated as linked loci. Finally, environmental information was included in one analysis as continuous characters in a trait partition. Each marker was assigned its own substitution model and clock rate. Substitution models for sequence data were determined by jModelTest [47]. Binary data had a simple evolution model, and environmental traits were given a homogeneous Brownian model of evolution. For the molecular dating, a strict clock model was used, with a fixed rate of 4.2E^−9^ substitutions per site per year [25] for the chloroplast sequences. The rest of the substitution rates were estimated relative to the fixed rate. In all reconstructions, the trees were linked and the analysis was run for 100 million generations. Results were analysed with Tracer 1.5 and TreeAnnotator 1.7.3 [45], using mean heights and maximum credibility options. Only those samples for which we had information form all sets of markers were used.

Additionally, we performed an AGglomerative NESted cluster analysis (agnes) of the environmental information to be able to compare the structure of this data with, the topologies obtained with phylogenetic methods. This method is based on a distance matrix of the two principal components per individual, and finds clusters with the highest agglomerative coefficient (AC). We used a euclidean distance matrix, and an UPGMA clustering method, using the *agnes* option implemented by the *cluster* package in R (http://cran.r-project.org/web/packages/cluster/index.html).

## Results

### Niche Divergence and Ecological Niche Modeling

**Figure 1:**
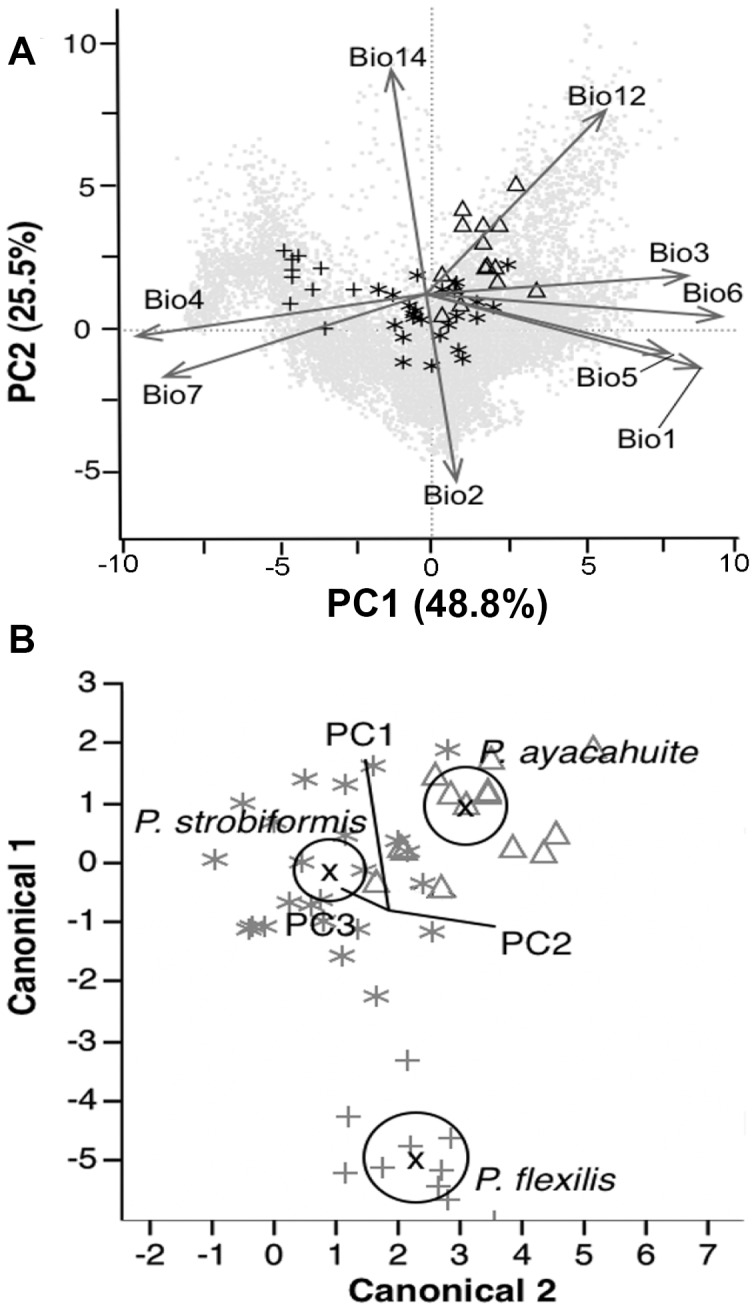
Environmental characterisation of the species complex. (A) Principal component analysis (PCA) of climatic variables for each species and over the entire climate availability (grey dots). The contribution of each component to the total variation is denoted in each axis. Arrows indicate the variables with a higher factor loadings for each component: Bio1: Annual Mean Temperature; Bio2: Mean Diurnal Range; Bio3: Isothermality; Bio5: Max. Temperature of Warmest Month; Bio6: Min. Temperature of Coldest Month; Bio7: Temperature Annual Range; Bio12: Annual Precipitation; Bio14: Precipitation of Driest Month. (B) Canonical analysis of the 3 principal components for the 3 species. Circles represent 95% confidence intervals.

The PCA showed that 74.3% of the variation was explained by the first two components and that each species had mostly its own environmental space with some overlap among species pairs (Fig. 1). The variables that had a higher contribution to the first component (PC1) were those related to mean temperature, temperature seasonality and isothermality, and temperature in the coldest month. The variables that contributed more to the second component (PC2) were the precipitation in the coldest month, mean diurnal temperature range and annual precipitation (Fig. 1a). The MANOVA analysis showed significant differences among species (Wilk’s lambda p<0.0001, d.f. 6/88), as well as each of the orthogonal contrast analyses for *P. flexilis* vs. *P. ayacahuite-P. strobiformis* (F-test, d.f. 3/44; p<0.0001), and *P. strobiformis* vs. *P. ayacahuite* (F-test, d.f. 3/44; p<0.0001). The canonical analysis also showed that all three species had significant differences in environmental variables. *P. ayacahuite* variance is more influenced by PC2, and *P. strobiformis* by PC1 and PC3. *P. flexilis* was very different from the other two species (Fig. 1b). The PC1 and PC2 were negatively correlated with latitude (r^2^ = 0.813, p<0.0001; r^2^ = 0.411, p<0.0001 respectively) and positively correlated with longitude (r^2^ = 0.477, p<0.0001; r^2^ = 0.3313, p<0.0001).

**Figure 2:**
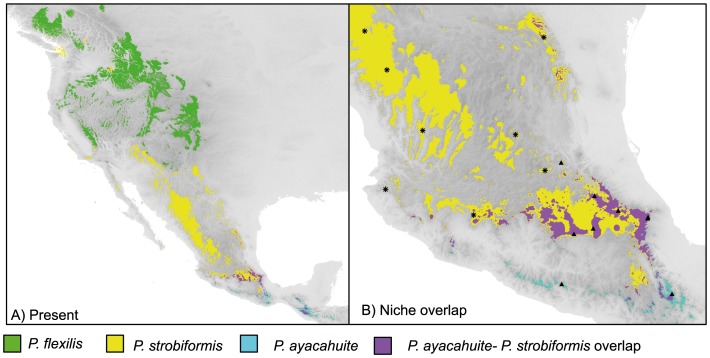
Ecological Niche Models for the present conditions. (A) Colours represent the range of *P. flexilis* (green), *P. strobiformis* (yellow) and *P. ayacahuite* (cyan), with range overlaps shown in magenta. Zoom to the contact zone; (B) of the TMVB and SMOR with presence sites for *P. ayacahuite* Δ and *P. strobiformis* *.

The ecological niche models had a good power of occurrence prediction (AUC >0.90), and agree with the species known distributions (Fig. 2a). Potential range overlap between *P. strobiformis* and *P. ayacahuite* is readily apparent (Fig. 2b). The overlap occurs in the TMVB and the Sierra Madre Oriental and Chiapas Highlands, but not in the Sierra Madre Occidental and Sky Islands. The range overlap for *P. flexilis* with the other two species was negligible. The LGM distribution showed mayor range displacements to the South of *P. flexilis* and *P. strobiformis,* and a considerable range overlap between *P. strobiformis* and *P. ayacahuite* (Fig. S1 in File S1).

**Figure 3:**
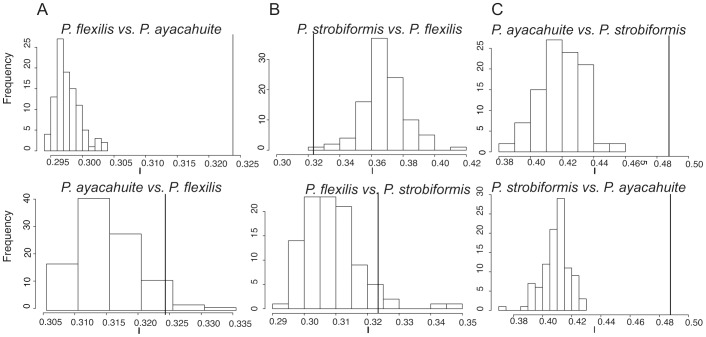
Histograms of the background (random) distribution of *I* values (niche similarity statistic) of pairwise species comparisons (A-C) after 100 replicates, which corresponds to a p>0.01. Observed *I* values for each pairwise comparison are indicated by the vertical line.

The background test showed that the *I* value (similarity) of *P. flexilis* was not larger than expected by chance in two-way comparisons with *P. strobiformis* (p>0.01; Fig. 3). On the other hand, the comparison *P. flexilis-P.ayacahuite* had a value of *I* significantly larger than expected at random (p<0.01), but the comparison *P. ayacahuite-P. flexilis* was not significant, suggesting an asymmetric niche similarity. The value of niche similarity (*I*) of *P. ayacahuite* and *P. strobiformis* was higher than expected by chance in both comparisons (p<0.01; two-tailed test for values of niche similarity *I*; Fig. 3).

### Genetic Differentiation and Phylogenetic Analyses

Of the chloroplast markers considered, only *atpB-rbcL* (GenBank accessions: FJ529007–FJ529013) intergenic spacer and the *trnL-trnF* intergenic spacer (GenBank accessions: FJ528997–FJ529006) were polymorphic. The intron of *trnG* (GenBank accessions: FJ487564–FJ487568) and the intergenic spacer *trnG-trnS* (GenBank accessions: FJ487569–FJ487573) were monomorphic for the three species under study and therefore not included in the analyses. The intron 1 of *nad5* was also monomorphic, except for a microsatellite region in the sequence that had length polymorphism, but it was not included in the dataset. The intron 4 of *nad5* (*nad5d*) was polymorphic and all analyses were carried out with these sequences (JN050888–JN050949). Sequence alignments of each data set were deposited in the Dryad Repository: http://dx.doi.org/10.5061/dryad.3ng7p

The number of haplotypes and segregating sites in the three species was low for both cytoplasmic markers (Table 1), but haplotype and nucleotide diversity was higher for *P. flexilis* chloroplast markers. The most frequent chloroplast haplotype was shared among all three species, but only *P. flexilis* and *P. strobiformis* had private haplotypes. Also, only *P. flexilis* showed significant genetic differentiation with *P. ayacahuite* and *P. strobiformis* for the three genetic differentiation metrics used (Table 1). These last two species shared more haplotypes (2) and had lower genetic variation and no significant genetic differentiation. The mitochondrial marker (*nad5d*) showed an opposite pattern of differentiation to that of the chloroplast, with higher genetic diversity in *P. strobiformis* and non-significant genetic differentiation with *P. flexilis* (Table 1). *P. ayacahuite* showed significant genetic differentiation with *P. strobiformis* and *P. flexilis* (Table 1).

**Table 1:** Genetic diversity and genetic differentiation within and between species.

*atpb-rbcL/trnL-trnF*	*n*	*h*	*S*	*H*_D_	π	θ_W_	*H*_ST_	*F*_ST_	*S*_nn_
*P. strobiformis*	22	4	3	0.2597	0.0003	0.0008			
*P. ayacahuite*	19	2	1	0.1052	0.0001	0.0003			
*P. flexilis*	16	4	3	0.7	0.0009	0.0009			
*P. strobiformis-P.ayacahuite*	41	4	3	0.1866	0.0002		−0.0117	−0.0259	0.4641
*P. strobiformis-P. flexilis*	38	7	6	0.4893	0.0006		**0.0987**	**0.1047**	**0.1047**
*P.ayacahuite-P. flexilis*	35	5	4	0.4403	0.0005		**0.151**	**0.1206**	**0.1206**
*nad5d*									
*P. strobiformis*	26	2	3	0.48	0.0016	0.001			
*P. ayacahuite*	20	2	2	0.3947	0.0015	0.001			
*P. flexilis*	9	1	0	0.0	0.0	0.0			
*P. strobiformis-P.ayacahuite*	46	3	2	0.5517	0.002		**0.1962**	**0.3493**	**0.6265**
*P. strobiformis-P. flexilis*	35	3	2	0.3832	0.0013		0.0302	0.2457	0.6439
*P. flexilis-P.ayacahuite*	29	2	2	0.5172	0.002		**0.4505**	**0.7368**	**0.7613**

Numbers in bold indicate statistically significant values at 99% confidence level.

*n*: number of sequences; *h*: number of haplotypes; *S*: segregating sites; *H*_D_: haplotype diversity; π: nucleotide diversity; θ_W_: Watterson estimate for population mutation rate; *H*_ST_: haplotype population differentiation; *F*_ST_: genetic differentiation; *S*_nn_: nearest neighbour statistic.

**Figure 4:**
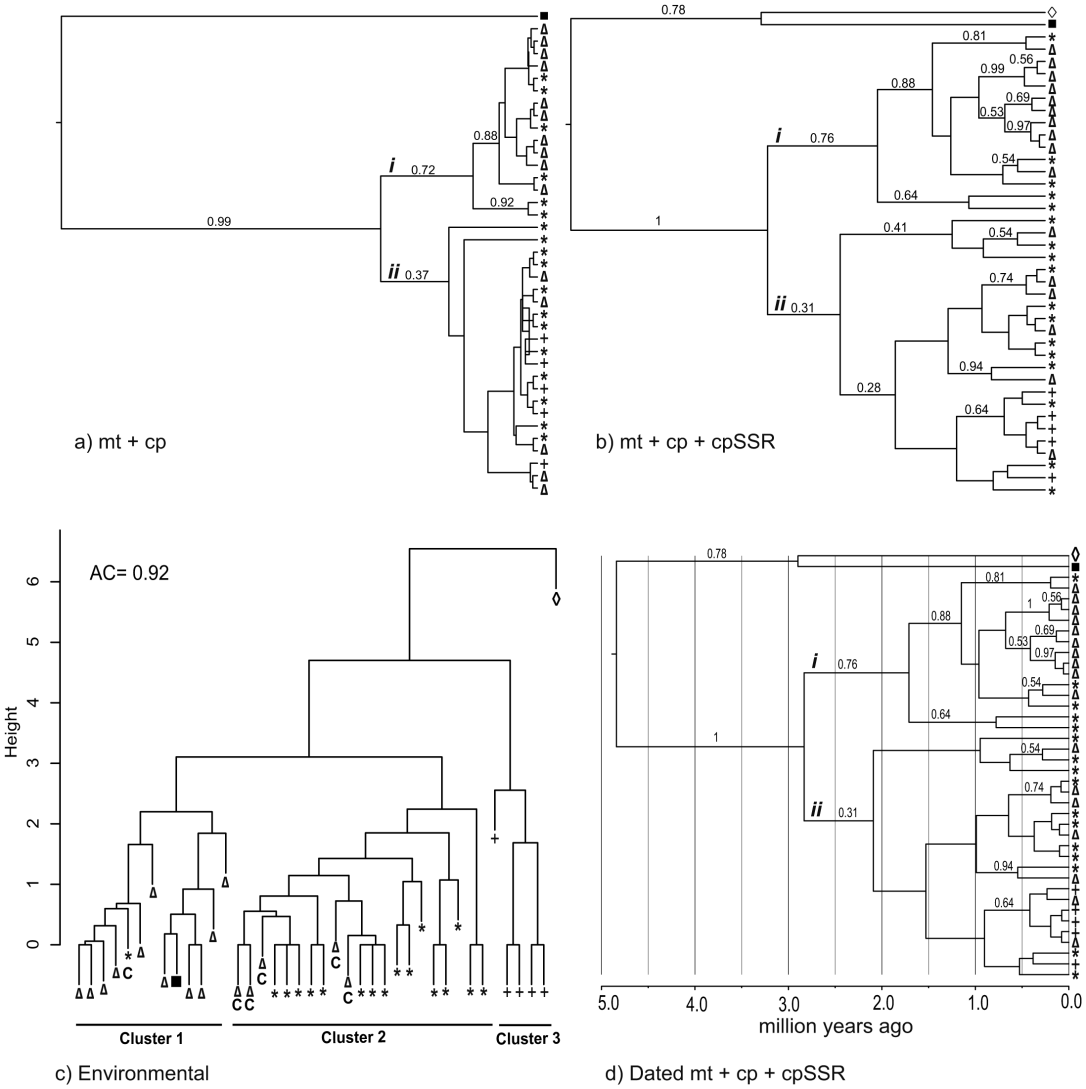
Bayesian genealogical reconstruction using different combinations of data. (A) Mitochondrial and chloroplast intergenic sequences; (B) Sequence data plus chloroplast microsatellites (cpSSR) coded as binary data; (C) Agglomerative nested cluster analysis of environmental information (principal components, C: contact zone; see Figs. 1 and 2); (D) Dated multilocus genealogy. Species key: Δ *Pinus ayacahuite*; * *Pinus strobiformis*; + *Pinus flexilis*; *▪ Pinus strobus*; ◊ *Pinus lambertiana*.

Different combinations of markers and traits produced trees with different levels of support for interspecific relationships. Chloroplast and mitochondrial sequence markers had the least resolution, only being able to recover a clade with mainly *P. ayacahuite* samples, but with low posterior probability (Fig. 4a). The addition of cpSSRs to the reconstruction helps resolve some tip nodes, but deeper relationships remain with low support (Fig. 4b). However, the combination of sequence data and environmental traits produced high support for the *P. ayacahuite* clade and some tip branches, although the *P. strobiformis*-*P. flexilis* clade remained with a low support value (Fig. S2 in File S1). The *agnes* analysis including environmental traits and genetic data shows a pattern of complete differentiation of *P. flexilis* (cluster 3, Fig. 4c), and two other clusters of mainly *P. ayacahuite* (cluster 1), and mainly *P. strobiformis* (cluster 2). All the samples that clustered in the “wrong” group (*P. strobiformis* and *P. ayacahuite*) came from sites found in the inferred contact zone of the Trans-Mexican Volcanic Belt (Fig. 2b), and therefore have intermediate environmental values. The molecular dating was performed for the genetic data. The divergence of the species complex using as outgroups *P. lambertiana* and *P. strobus*, corresponds to the late Pliocene-Late Pleistocene transition (Fig. 4d; 2.8 Mya 95% HPD 0.21–7.4 Ma). No other divergence dates were considered, due to the low support values of some branches.

**Figure 5:**
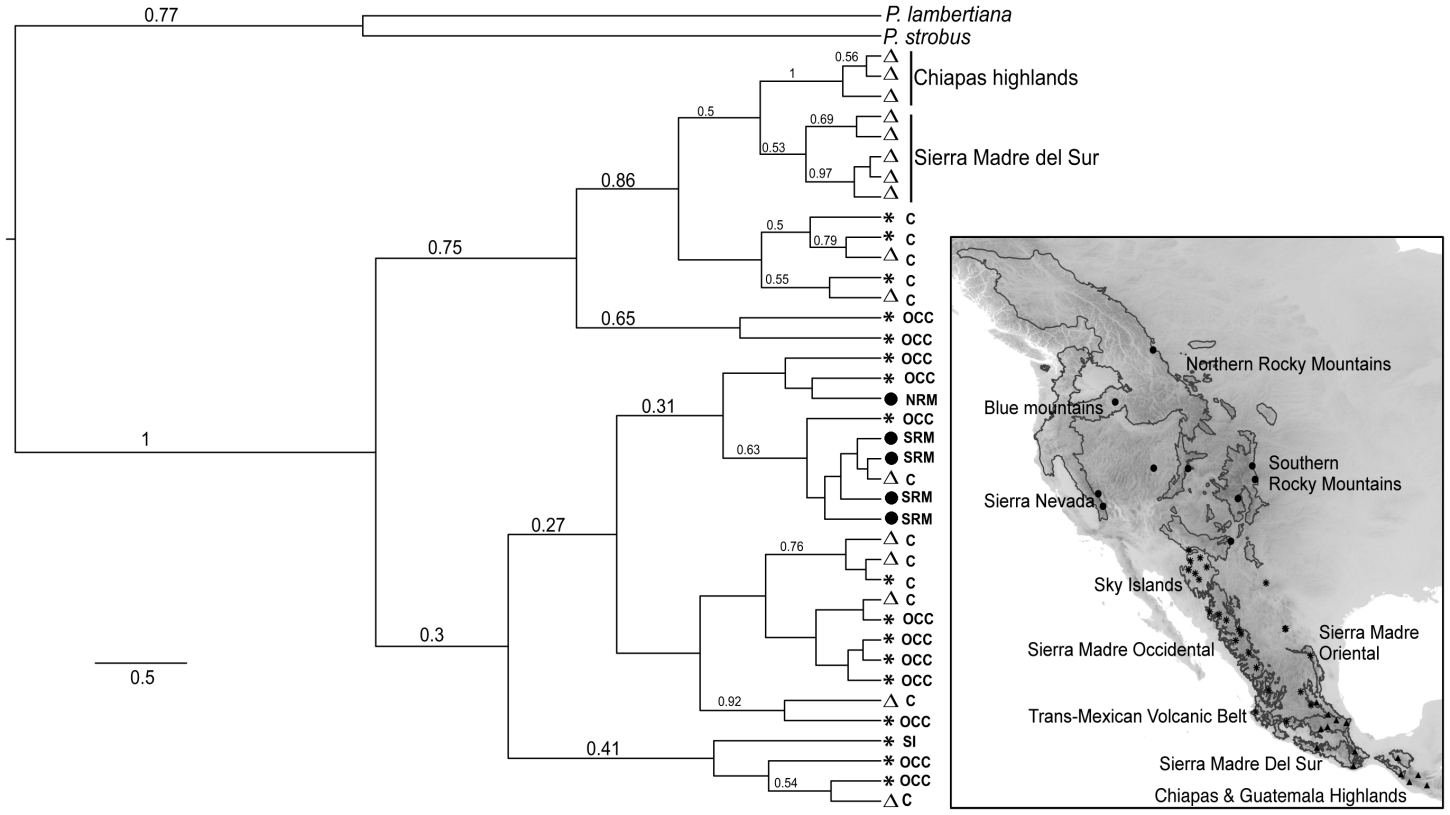
Total evidence tree with highlighted phylogeographic structure. Letter codes next to the species symbol denote the geographical region of each sample. OCC: Sierra Madre Occidental; NRM: Northern Rocky Mountains; SRM: Southern Rocky Mountains; SI; Sky Islands; C: Contact zone comprised of the Sierra Madre Oriental and Trans-Mexican Volcanic Belt (See Fig. 2). Species key: Δ *Pinus ayacahuite*; * *Pinus strobiformis*; • *Pinus flexilis*; ▪ *Pinus strobus*; ◊ *Pinus lambertiana*.

Despite the low support of some tip branches and shared polymorphism in cytoplasmic markers, some phylogeographic structure can be observed in the phylogenetic reconstruction (Fig. 5). All samples from the Chiapas highlands and Sierra Madre del Sur group together with a high posterior probability, and form a sister group with samples from the contact zone. The other poorly supported clade includes individual from *P. flexilis*, *P. strobiformis* and the contact zone, with no phylogeographic structure.

## Discussion

### Niche Divergence and Ecological Niche Modeling

PCA and MANOVA analyses showed that each species has its own ecological niche (Fig. 1). From the PCA results, the variables related to mean temperature and temperature range contributed more to PC1. The three species were distributed in a gradient from higher mean temperature (*P. ayacahuite*) to lower mean temperature for *P. flexilis*, with intermediate values for *P. strobiformis*, which is congruent with a latitudinal cline (Fig. 1). *P. flexilis* and *P. strobiformis* have wider annual and diurnal fluctuations in temperature (isothermality; Fig. 1), whereas for *P. ayacahuite* conditions are more uniform. The minimum temperatures in the coldest month are observed in the P. *flexilis* populations, and the maximum temperatures during the warmest month are observed for *P. strobiformis*.

The PC2 has higher contribution from precipitation related variables (Fig. 1). The gradient of annual precipitation goes from higher precipitation for *P. ayacahuite* to lower precipitation for *P. flexilis*, with intermediate levels for *P. strobiformis*. However, the amount of precipitation *P. ayacahuite* receives during the winter is lower, that that of the other two species (t-test p<0.05). Even though the mean annual temperature for *P. strobiformis* is only slightly lower than for *P. ayacahuite*, the extreme minimum temperatures for part of the range of *P. strobiformis* can be much lower. Both the SMOR and SMOCC can have extreme low temperatures during the winter months, reaching −20°C at night but up to 15°C during the day in some areas of the SMOCC, whereas *P. ayacahuite* populations only reach −5°C (Sistema Meteorologico Nacional; http://smn.cna.gob.mx). These extreme diurnal fluctuations could mean higher cold stress for *P. strobiformis* due to repeated freezing and thawing cycles.

The niche differentiation in space can also be seen in the ENM projection (Fig. 2a), where *P. flexilis* has virtually no overlap with *P. strobifomis* or *P. ayacahuite*. However, there is a broad area of overlap between *P. strobiformis* and *P. ayacahuite* mainly in the TMVB, an area that has already been identified as a contact zone for other temperate species (Fig. 2b; [48]). However, is important not to forget that ENMs are a simplification of the potential niche of a species and therefore, an over-prediction. The actual range of both *P. strobiformis* and *P. ayacahuite* in the TMVB is more patchy and fragmented, so factors other than climate could determine their distribution ([14]; pers. obs. AML). In the present range, the closest populations of both species in the TMVB are 56 km apart, but previous analyses with chloroplast microsatellites did not show evidence of gene flow (shared haplotypes and low genetic differentiation) between these two populations [28]. The evidence of gene flow between *P. strobiformis* and *P. ayacahuite* in the present study involves populations that are farther apart (at least 400 km), beyond the distance that pollen can disperse [49]. However, our distribution model for the LGM (Fig. S1 in File S1) suggests that the range of *P. strobiformis* and *P. ayacahuite* was contiguous, with a broad potential contact zone. This evidence, together with morphological evidence which identifies the individuals of SMOR as *P. strobiformis* [14], suggests that the hybridisation is not contemporary and/or similarity is due to retention of ancestral polymorphisms, and also agrees with the retention of *P. strobiformis*-like morphology. Similar cases have been reported among eight European white oak species [50] and between two chloroplast haplotypes of *P. lambertiana* [21] where there was an organelle capture after hybridisation, but retaining the morphology of one of the parental species. In this case, the populations of *P. strobiformis*-like individuals from SMOR seem to have captured both chloroplast and mitochondria from *P. ayacahuite*. However, the degree of introgression between these two taxa can only be determined with multiple nuclear loci. On the contrary, the populations from SMOCC seem to be evolving separately (Fig. 2a).

While the multivariate analyses of environmental conditions and little niche overlap show niche differentiation for the three species, this type of analyses do not take into account the available background environment of lineages, which could bias the results towards niche differentiation [32], [36], [51]. To further evaluate the niche similarity, we performed a background test. Our results confirm the niche divergence of *P. flexilis* with *P. strobiformis* as the *I* value is not larger than expected by chance (Fig. 3). This rejects the idea that *P. flexilis* forms a cline with *P. strobiformis,* as has been suggested by other authors [14], [52], [53].

Since there is no predicted niche overlap of *P. flexilis*, recurrent gene flow seems unlikely with the other two species of the complex, thus we can assume that the shared polymorphism is due to common ancestry. This observed niche divergence could suggest ecological speciation of *P. flexilis* [32]. However, the background test showed that *P. flexilis* could predict part of the niche of *P. ayacahuite* (p<0.01; Fig. 5), but *P. ayacahuite* could not predict the niche of *P. flexilis* (p>0.01). In this case we can say that the niche of *P. ayacahuite* is nested within the *P.flexilis* niche, and that there is certain degree of niche conservatism.

The background test for *P. strobiformis* and *P. ayacahuite* (Fig. 3), showed significant niche similarity, despite the fact that their current observed distribution is not sympatric. This niche conservatism could suggest allopatric speciation, with the areas of unsuitable habitat acting as dispersal barriers [30]. However, the background test also evaluates the degree in which the niche model of one species can predict the niche of it’s sister species [36]. The niche model for *P. strobiformis* can predict most of the range of *P. ayacahuite*, but the model of *P. ayacahuite* does not predict the distribution of *P. strobiformis* in the SMOCC and Sky Islands (Fig. 2). This suggests that *P. strobiformis* has a broader niche and ecological tolerance than *P. ayacahuite*, and thus the niche of the latter is also nested. Niche differentiation could be occurring in the populations from the Northwestern part of the range, but the degree of overlap in the TMVB is perhaps too large for the background test to discriminate this. This results is concordant with the East-West differentiation found with chloroplast microsatellites [28], where the populations from the SMOCC and Sky Islands share few haplotypes with *P. ayacahuite* and have many unique haplotypes in high frequency.

### Genetic Differentiation and Phylogenetic Analyses

Overall, the level of polymorphism found in the species complex was low and several haplotypes were shared among species. This low polymorphism at the chloroplast level was already reported in literature, even for whole chloroplast sequences (π*_ayacahuite-flexilis_* = 0.000165; Whittall et al., 2010). The mitochondrial markers also showed similar levels of polymorphism (number of haplotypes) reported for *P. albicaulis* (3 haplotypes) and *Picea chihuahuana* (2 haplotypes), but lower than those reported for hard pines and other conifers [54], [55]. The general pattern for both genomes was of shared polymorphism among the members of the species complex for chloroplast markers and with other white pines for mitochondrial markers, consistent with what was reported by other authors [27], [28], [56], [57]. The reason behind shared ancestral polymorphism could be that coalescent times are older than the divergence of the species, due to large effective population sizes and recent speciation events, or a low mutation rate in the case of the mitochondria [58]–[60].

The pattern of genetic differentiation among species was incongruent between chloroplast and mitochondrial markers. Both sequence based (*S*_nn_) and haplotype based (*H*_ST_ and *F*_ST_) estimates of genetic differentiation for the chloroplast showed a clear differentiation of *P. flexilis* (Table 1), however, for the mitochondrial marker, it is *P. ayacahuite* the species that is significantly different, suggesting lineage sorting for *P. ayacahuite* populations. The genetic differentiation of *P. ayacahuite* with the microchondrial marker is to be expected due to its maternal inheritance (seed) and less dispersal capacity than pollen.

Shared polymorphism is apparent in the different reconstructions (Fig. 4). All analyses recover two main clades, with varying degrees of support. Clade *i* is comprised mostly *P. ayacahuite* individuals, and a few *P. strobiformis* individuals from the contact zone. The second clade (*ii*) is comprised of *P. flexilis* and *P. strobiformis* individuals, with a few individuals of *P. ayacahuite* from the contact zone. The common geographic origin of all incongruent samples suggests that hybridisation has been a common phenomenon in the Trans-Mexican Volcanic Belt and Sierra Madre Oriental. Hybridisation and introgression are also supported by morphological studies and chloroplast microsatellites in the Eastern populations of *P. strobiformis* [28], [61]. Evidence of genetic connectivity of these two regions was also detected in populations of the fungal endophyte *Lophodermium nitens*, an obligate commensal of white pines [62]. This contact zone identified with molecular markers, corroborates out finding of an ecological overlap (Fig. 2b and Fig. 5), and blurs the boundaries between *P. strobiformis* and *P. ayacahuite*, making it difficult to determine if it is due to parapatric speciation or a secondary contact zone. To distinguish between these two processes, we would suggest the addition of nuclear markers and a different sampling strategy along the climatic cline.

The lack of any apparent phylogeographic structure in clade *ii* suggests incomplete lineage sorting between *P. flexilis* and *P. strobiformis*, more than recent gene flow. The individuals within clade *ii* share a single mitochondrial haplotype and closely related chloroplast haplotypes, but the geographic distances separating populations are too great to attribute these similarities to recent gene flow (Fig. 5). Additionally, *P. flexilis* has a distinctly different ecological niche (Fig. 3 and Fig. 4c), which could make the survival of any hybrids unlikely.

The use of different types of data yielded different results in the phylogenetic reconstruction. The inclusion of cpSSR data increased the resolution at the tips of the tree, but did not increase the support of internal branches. This is due to their higher mutation rate and the presence of homoplasy, that can obscure old relationships [28], [63], [64]. The inclusion of environmental information in the phylogenetic reconstruction did increase the support of clade *i*, and even of the weakly supported clade *ii* (Fig. S2 in File S1), but did not improve the resolution of tip branches. This could be due to the low polymorphism of the data, and the lack of correlation between the cytoplasmic markers and environment at the intraspecific level. However, environmental data for each individual is informative enough to distinguish clusters that roughly correspond to the three species (Fig. 4c).

The low polymorphism in the markers used suggests recent divergence. This is confirmed by the Pliocene-Early Pleistocene divergence of the species complex (Fig. 4d; [65]). This divergence date is also congruent to the divergence dates estimated for *L. nitens* [62], and for insects, reptiles and plants with similar distributions [66]–[69]. This highlights the importance that Pleistocene glaciations had in the evolution of taxa of temperate affinities in the subtropics.

## Conclusions

Our results show that the *P. strobiformis-P. flexilis-P. ayacahuite* species complex is indeed a group of recent origin, where each species exhibits ecological differentiation. Neutral genetic markers do not reflect this differentiation entirely, due to hybridisation and incomplete lineage sorting. This suggests that niche divergence and local adaptation have occurred at a faster pace than the lineage sorting of neutral markers. Genetic markers place *P. flexilis* and *P. strobiformis* as more closely related to each other than *P. ayacahuite*, but *P. strobiformis* and *P. ayacahuite* are more ecologically similar than *P. flexilis*. This contrasting pattern suggests that different evolutionary forces are at play: natural selection as the cause of ecological differentiation, and genetic drift and gene flow are behind the genetic structure of cytoplasmic markers.

The results presented here point towards ecological speciation as possible cause of the high diversity of pines in North America, and highlight the necessity of studying the genes involved in local adaptation in more species, to better understand the process.

## Supporting Information

File S1 **Supporting information for: Niche divergence versus neutral processes: Combined environmental and genetic analyses identify contrasting patterns of differentiation in recently diverged pine species. Table S1.** Coordinates used in Ecological Niche Modeling. **Table S2.** Sampling information for *Pinus flexilis.* **Figure S1.** Ecological Niche Model under Last Glacial Maximum conditions. **Figure S2.** Phylogenetic reconstruction using environmental information.
(PDF)
