# Supplementary material for: Niche Divergence versus Neutral Processes: Combined Environmental and Genetic Analyses Identify Contrasting Patterns of Differentiation in Recently Diverged Pine Species
Source: PLoS One. 2013 Oct 29;8(10):e78228. doi: 10.1371/journal.pone.0078228 (PMC3812143; doi:10.1371/journal.pone.0078228)
Supplement: File S1 — Supporting information for: Niche divergence versus neutral processes: Combined environmental and genetic analyses identify contrasting patterns of differentiation in recently diverged pine species. Table S1. Coordinates used in Ecological Niche Modeling. Table S2. Sampling information for Pinus flexilis. Figure S1. Ecological Niche Model under Last Glacial Maximum conditions. Figure S2. Phylogenetic reconstruction using environmental information. (PDF) [file pone.0078228.s001.pdf]

Supplementary material for the manuscript: **Niche divergence versus neutral processes: Combined environmental and genetic analyses identify contrasting patterns of differentiation in recently diverged pine species.**

Alejandra Moreno-Letelier, Alejandra Ortiz-Medrano and Daniel Piñero

Table S1.- Coordinates used in ENM modeling of the *Pinus strobiformis, Pinus flexilis* and *Pinus ayacahuite* species complex.

Species	Longitude	Latitude
Pstrobiformis	-100.222	24.8882
Pstrobiformis	-104.9489	23.9215
Pstrobiformis	-105.6197	25.0749
Pstrobiformis	-106.5014	26.9419
Pstrobiformis	-107.5836	27.6888
Pstrobiformis	-108.2018	28.1658
Pstrobiformis	-109.0274	28.3598
Pstrobiformis	-104.9917	20.3709
Pstrobiformis	-101.0725	21.9936
Pstrobiformis	-110.8661	31.699
Pstrobiformis	-110.7093	32.41
Pstrobiformis	-110.851	33.2894
Pstrobiformis	-109.2752	31.9347
Pstrobiformis	-110.2987	31.4198
Pstrobiformis	-109.8659	32.653
Pstrobiformis	-102.3813889	27.035
Pstrobiformis	-102.3386667	19.60805556
Pstrobiformis	-100.1849167	20.92927778
Pstrobiformis	-102.4470639	26.99946944
Pstrobiformis	-106.2834111	26.60074722
Pstrobiformis	-103.8727778	22.11722222
Pstrobiformis	-104.1083333	30.65566667
Pstrobiformis	-106.7578333	26.08131667
Pstrobiformis	-109.9583333	30.94166667
Payacahuite	-98.73591667	19.21036111
Payacahuite	-97.98225	19.73813889
Payacahuite	-98.69441667	20.17888889
Payacahuite	-99.70091667	21.16863889
Payacahuite	-99.31527778	19.04833333
Payacahuite	-90.83333333	15.38333333
Payacahuite	-92.58483333	16.72783333
Payacahuite	-92.33061111	15.42872222
Payacahuite	-99.68555556	17.55655556
Payacahuite	-96.36136111	17.26477778
Payacahuite	-96.46838889	16.10744444

Payacahuite	-97.09444444	19.50833333
Payacahuite	-97.08333333	19.55
Payacahuite	-90.0625	14.55416667
Payacahuite	-91.59166667	14.82166667
Pflexilis	-110.9272222	39.76277778
Pflexilis	-113.9666667	49.21666667
Pflexilis	-106.5666667	37.38333333
Pflexilis	-105.3031667	39.9835
Pflexilis	-113.9169	39.8
Pflexilis	-117.275	45.0578
Pflexilis	-107.1758333	33.99138889
Pflexilis	-105.0605556	38.89555556
Pflexilis	-118.3358	36.7756
Pflexilis	-118.767502	37.767502

Table S2.- Information of the sampling localities for *Pinus flexilis*.

Locality	Country	Coordinates
Shulman Grove White Mnts., Inyo CA	USA	37˚ 35' 00'' N, 118˚16'03'' W
Canyon Recreation Area, Carbon UT	USA	39˚45'46'' N, 110˚55'38'' W
Waterton Lakes Natl. Park, Alberta	Canada	49˚13' N, 113˚58' W
Rio Grande Natl. Forest, Conejos Co	USA	37˚23' N, 106˚34' W
Cunén, El Quiché Department	Guatemala	15˚ 23' 00'' N, 90˚ 50' 00'' W

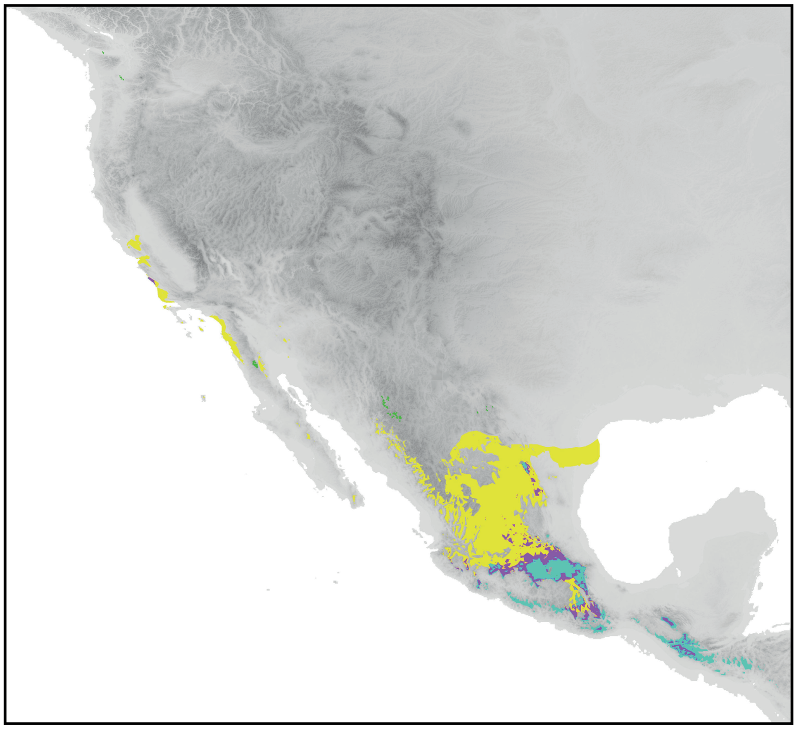

P. flexilis — P. strobiformis — P. ayacahuite — P. ayacahuite- P. strobiformis overlap

Figure S1.- Environmental niche model under Last Glacial Maximum conditions. Different colours represent the predicted range of each species. Range overlap of *P. ayacahuite* and P. *strobiformis* is represented in magenta.

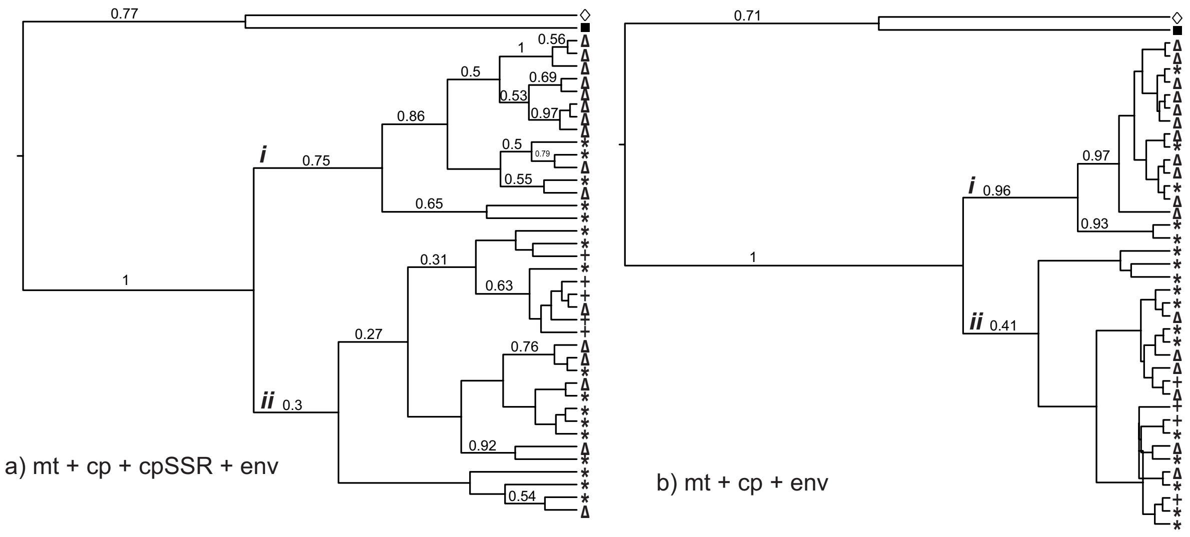

Figure S2.- Phylogenetic reconstructions using genetic data and environmental information coded as traits: a) Mitochondrial and chloroplast sequences, chloroplast microsatellites with environmental data; b) Mitochondrial and chloroplast sequences with environmental data. Δ *Pinus ayacahuite*; + *Pinus flexilis*; * *Pinus strobiformis*
